# TachoSil^®^ as a ventricular sealant after glioma resection: A single-center retrospective cohort study

**DOI:** 10.1007/s10143-026-04484-7

**Published:** 2026-09-24

**Authors:** Matteo Maria Ottaviani, Giuseppina Bevacqua, Valentina Grespi, Carlo Conti

**Affiliations:** 1 Department of Neurosurgery, Università Politecnica delle Marche, Ancona, Italy; 2 Department of Neurosurgery, Santa Maria della Misericordia Hospital, Perugia, Italy; 3 Department of Neuroscience, Neurosurgery Unit, Santa Maria Hospital, Terni, Italy; 4 Laboratorio Cellule Staminali, Cell Factory e Biobanca, Santa Maria Hospital, Terni, Italy

**Keywords:** TachoSil^®^, Cerebral ventricles, Cerebrospinal fluid, Glioma, Ventricular closure, Case series, Neuro-oncology

## Abstract

Ventricular entry during resection of periventricular gliomas may increase the risk of cerebrospinal fluid (CSF)-related complications, while optimal management of ependymal defects remains uncertain. We evaluated the feasibility, short-term safety, and postoperative imaging characteristics of onlay TachoSil® ventricular sealing after supratentorial high-grade glioma resection, supported by a focused synthesis of the available neurosurgical literature. We retrospectively reviewed 32 consecutive adult patients undergoing WHO grade 3–4 glioma resection between January 2024 and January 2026 in whom an intraoperative ventricular opening was sealed with a TachoSil® patch placed over the ependymal defect. CSF-related subcutaneous collection or wound leakage occurred in 5/32 patients (15.6%) within 30 days; four were managed conservatively and one required temporary lumbar drainage. No patient developed meningitis or ventriculitis, shunt-dependent hydrocephalus, early patch-related obstruction requiring reoperation, or need for permanent CSF diversion. Serial MRI through 3 months demonstrated a thin laminar interface centered at the ventricular repair site, with predominantly preserved CSF-like intraventricular signal and no patch displacement, free intraventricular material, CSF-pathway obstruction, progressive ventriculomegaly, or definite radiological migration. These findings support the technical feasibility and short-term safety of onlay TachoSil® ventricular sealing and suggest that the patch may provide a stable neomembrane-like interface between the resection cavity and ventricular system. However, the absence of a control group, the 15.6% rate of CSF-related collections, and limited follow-up preclude conclusions regarding comparative efficacy or rare delayed complications. Prospective multicenter studies with standardized closure endpoints and longer follow-up are warranted.

## Highlights

Onlay TachoSil^®^ ventricular sealing was feasible in 32 consecutive supratentorial glioma resections with intraoperative ventricular entry.

Subcutaneous CSF leakage occurred in 5/32 patients and was managed conservatively in 4 patients and with lumbar drainage in 1 patient.

No meningitis, ventriculitis, pseudomeningocele, shunt-dependent hydrocephalus, patch-related obstruction, or permanent CSF diversion were observed.

Serial MRI showed a thin ventricular repair interface separating the resection cavity from predominantly CSF-like intraventricular signal.

## Introduction

Postoperative CSF leakage remains a major driver of morbidity after cranial and spinal procedures requiring dural opening [4, 16, 36]. Persistent leakage is associated with meningitis and other infections, wound dehiscence and pseudomeningocele formation, intracranial hypotension with orthostatic headaches and subdural collections, longer hospitalization, and increased costs [16]. Adjunctive sealants and dural sealant patches have therefore been introduced to complement sutured closure and to facilitate watertight repair in anatomically constrained operative corridors [4, 10, 13].

TachoSil^®^ is a ready-to-use, bioabsorbable fibrin sealant patch designed to achieve local hemostasis and sealing upon contact with blood or physiological fluids [4]. It is used intraoperatively as an adjunct when standard surgical techniques are insufficient, to improve hemostasis and promote tissue sealing [8, 20]. TachoSil^®^ was developed as a next-generation evolution of the earlier fibrin-coated collagen patches TachoComb and TachoComb H and subsequently translated to neurosurgery as an adjunct for watertight dural repair to reduce CSF leaks [13, 24, 28, 34]. It received its first European marketing authorization on June 8, 2004, and initial U.S. FDA approval on April 5, 2010. Across neurosurgical applications, TachoSil^®^ is used either as an adjunct to primary suturing or as a primary sealant when suturing is impractical, including multilayer reconstructions and sutureless ‘sandwich’ techniques [22, 23, 29, 32, 35].

Ventricular opening during periventricular tumor resection is frequent [20] and, although small openings were historically often left unsealed [14], ventricular breach may disrupt CSF dynamics and contribute to CSF fistula or pseudomeningocele, hydrocephalus, and meningitis [15]. Ventricular closure is therefore a distinct subdomain of CSF leak prevention because the goal extends beyond preventing extracranial CSF egress to restoring an effective intraventricular barrier [14, 16]. Failure to seal the ventricular system can allow migration of hemostatic material or tumor debris into CSF pathways, increase the risk of obstructive hydrocephalus, and preclude safe implantation of carmustine wafers [16, 18]. Early work with fibrin adhesive suggested that sealing the transcortical tract after ventricular entry can prevent postoperative pseudomeningoceles [1, 4] and TachoSil^®^ has since been proposed as an “artificial ependyma” placed over ependymal defects to separate the ventricular lumen from the resection cavity [13]. Here we report a single-center uncontrolled retrospective case series of 32 supratentorial glioma resections in which ventricular opening occurred to describe feasibility, postoperative imaging characteristics, and short-term safety of TachoSil^®^ in ventricular closing, supported by a focused synthesis of the available evidence on TachoSil^®^ ventricular repair.

## Materials and methods

### Study design and ethics

This was an exploratory, single-center retrospective cohort study of consecutive supratentorial glioma resections in which intraoperative ventricular entry was sealed using an onlay TachoSil^®^ patch. The study was designed as a descriptive feasibility, short-term safety, and postoperative imaging report. It was not designed to test comparative efficacy, and the absence of an untreated or alternatively treated control group precludes attribution of outcomes to TachoSil^®^ itself. The study was conducted using routinely collected clinical and radiological data, which were analyzed in anonymized/de-identified form.

### Focused evidence synthesis

A focused evidence synthesis was performed to contextualize TachoSil^®^ handling, safety, and CSF-related outcomes in neurosurgery, with particular emphasis on ventricular applications and studies reporting postoperative CSF leakage, meningitis/ventriculitis, pseudomeningocele, hydrocephalus, patch migration, or imaging findings. This synthesis was used to support interpretation of the institutional cohort.

### Patient cohort

We retrospectively analyzed 32 consecutive adult patients who underwent supratentorial glioma resection among 221 performed at our institution from January 2024 to January 2026. In all cases of ventricular opening the defect was sealed with TachoSil^®^. Inclusion criteria were: adult patients undergoing supratentorial glioma resection during the study period; intraoperative ventricular opening; ventricular sealing with TachoSil^®^; and sufficient postoperative clinical/radiological follow-up to evaluate early CSF-related outcomes. Patients without ventricular entry, with documented previous ventricular entry for recurrent gliomas, and without sufficient follow-up data were excluded. No monetary incentives were offered for recruitment or retention. This surgical series includes only treated ventricular-entry cases; the retrospective dataset did not contain a reliable denominator of all glioma resections performed during the study period or of all ventricular openings managed without TachoSil^®^, and therefore systematic versus selective use of the technique cannot be assessed.

### Pre-intervention patient optimisation

Pre-intervention patient optimisation was performed as part of routine glioma surgery care and was individualized rather than dictated by the study. Before surgery, patients underwent neurosurgical and anesthesiology assessment, review of comorbidities and medications, coagulation/hematologic evaluation, and MRI-based operative planning. Antiplatelet/anticoagulant management, corticosteroids for edema, antiseizure medication, antibiotic prophylaxis, venous thromboembolism prophylaxis, fasting/nil-by-mouth status, and other stabilization measures were managed according to institutional protocols and patient-specific indications. No study-specific lifestyle, psychological, or monetary optimization program was used.

### Ventricular sealing technique

**Fig. 1:**
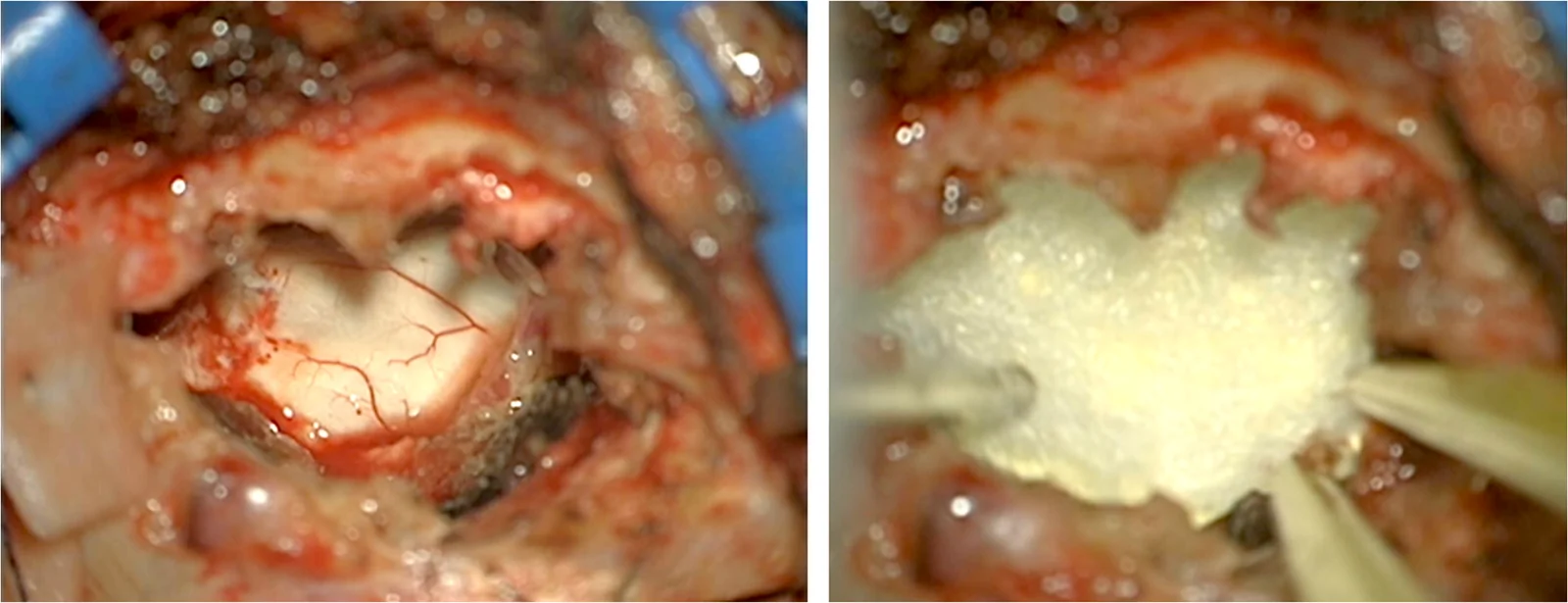
Occlusion of a surgical opening of the temporal horn of the lateral ventricle after resection of a recurrent malignant glioma (patient 7) using a TachoSil^®^ patch

Ventricular entry was recognized intraoperatively in all cases and was managed immediately after tumor removal and hemostasis. After completion of tumor resection and hemostasis, TachoSil^®^ was trimmed to overlap the margins of the ependymal defect by several millimeters and applied as an onlay patch with the active surface facing the ventricular opening (Fig. 1). Gentle compression using a moist neurosurgical patty was maintained for a standardized interval to promote adherence and sealing, and additional overlapping pieces of TachoSil^®^ were applied when the defect was broad, irregular, or located in a high-flow CSF region. The study intervention was restricted to the ventricular onlay patch: no alternative fibrin glue or other sealant was deliberately applied over the ependymal defect as the primary ventricular repair. Hemostatic agents placed within the resection cavity and any dural reinforcement used during closure were considered separate adjuncts and were not part of the ventricular sealing technique evaluated here. TachoSil^®^ was not systematically applied over the dura. A watertight dural closure was attempted in all cases using standard closure techniques, with grafts or dural sealant reinforcement left to the surgeon’s discretion when required by dural quality or closure tension. The integrity of the ventricular seal was assessed under irrigation and Valsalva maneuvers when feasible.

### Postoperative clinical and radiological assessment

After discharge, clinical follow-up was more intensive during the first postoperative month. Patients were scheduled for visits at the neurosurgical outpatient clinic on postoperative days 8 and 14 for wound inspection, evaluation of blood parameters, and clinical assessment. Patients underwent a scheduled outpatient clinical assessment at 1 and 3 months as part of routine follow-up. Clinical events occurring outside these scheduled assessments were collected only when documented during routine oncological/neurosurgical follow-up and were not considered systematically ascertained outcomes. All patients underwent early postoperative contrast-enhanced MRI within 72 h of surgery, at 1 and 3 months post-surgery.

Primary safety endpoints included postoperative pseudomeningocele requiring intervention, meningitis/ventriculitis, shunt-dependent hydrocephalus, early reoperation for suspected patch-related obstruction, and permanent CSF diversion. MRI was assessed for the relationship between the resection cavity, the ventricular lumen, and the linear ventricular repair interface. A dedicated safety review of the available MRI studies also searched for signs of patch migration, displaced intraventricular material, obstruction of CSF pathways, progressive ventriculomegaly, or delayed hydrocephalus.

### Migration-focused MRI safety review

Because this was a retrospective study, no additional research-specific MRI acquisition was performed. Instead, all routine postoperative MRI examinations obtained within 72 h and at 1 and 3 months were subjected to a dedicated migration-focused safety review, with the early study serving as the patient-specific postoperative baseline. Serial examinations were compared using the available multiplanar sequences to assess: (1) persistence and position of the expected thin laminar interface at the ependymal defect; (2) interval loss, discontinuity, folding, or displacement of that interface; (3) new free or displaced intraventricular patch-like material; (4) obstruction of the foramen of Monro, third ventricle, cerebral aqueduct, or other CSF pathways; and (5) new or progressive ventriculomegaly, transependymal CSF flow, or hydrocephalus. Definite migration was defined radiologically as interval displacement of patch-like material from the repair site into the ventricular lumen, particularly when accompanied by CSF-pathway obstruction or evolving ventricular enlargement. Because no validated MRI definition of TachoSil^®^ migration after ventricular sealing is currently available, these criteria were used as a pragmatic retrospective safety framework.

### Analysis

Analyses were descriptive. Continuous variables were summarized using medians and ranges when appropriate, and categorical variables were summarized as counts and percentages. No formal comparative hypothesis testing was planned because this was an uncontrolled case series focused on feasibility, short-term safety, CSF-related outcomes, and postoperative imaging characteristics. No contemporaneous or historical institutional control cohort was available for a reliable comparison. The study was also underpowered to exclude rare but clinically important adverse events such as patch migration, delayed obstructive hydrocephalus, or granulomatous reaction. This case series has been reported in line with the PROCESS 2025 guideline [46].

## Results

### Clinical cohort

A total of 18/32 procedures were performed for newly diagnosed glioma, and 14/32 for recurrent disease. All tumors were high-grade gliomas classified as WHO grade 3 or 4. For recurrent disease, this designation referred to patients previously operated on for a high-grade glioma; none had experienced ventricular opening during the prior procedure. Median age at surgery was 66.4 years (range 59–72), and 62.5% were male (20/32). Glioma lesions were predominantly periventricular, most commonly involving the frontal and parietal lobes (frontal 40%, parietal 25%, temporal 20%, occipital 10%, insular/deep structures 5%), with ventricular entry occurring most frequently at the frontal horn/body of the lateral ventricle (45%) and less commonly at the atrium/occipital horn (35%) or temporal horn (20%). Exact ventricular defect diameter was not measured prospectively. Operative notes qualitatively described some defects as broad, irregular, or high-flow, but no validated high-grade CSF-leak classification was available; therefore, the relationship between defect severity and postoperative CSF-related collections could not be tested.

### Deviation from the initial management plan

No case required abandonment of the planned onlay TachoSil^®^ ventricular sealing strategy or conversion to an alternative ventricular closure method. No other ventricular sealant was documented as the primary repair of the ependymal defect. In cases with broad, irregular, or higher-flow defects, the planned technique was adapted by adding overlapping TachoSil^®^ pieces and by using standard hemostatic measures within the resection cavity at the surgeon’s discretion; these adaptations were technical refinements of the planned closure rather than protocol deviations. No patch-related obstruction or early reoperation occurred.

### Postoperative course and MRI findings

Patients were advised to follow routine postoperative wound-care and activity instructions and attended the scheduled visits during the first postoperative month and the 3-month outpatient assessment; no participant was documented as lost to these scheduled follow-up evaluations. Clinical follow-up was more intensive during the first postoperative month, while the 3-month visit formed part of routine neuro-oncology and neurosurgical outpatient care.

CSF-related subcutaneous collection or wound leakage (pseudomeningocele) occurred in 5/32 patients (15.6%) during the first 30 postoperative days (median time to presentation 18 days). Four cases were managed with compressive measures and observation, whereas one case required temporary lumbar drainage. No patient required wound revision, surgical evacuation of a large pseudomeningocele, early reoperation for suspected TachoSil^®^-related obstruction, or permanent CSF diversion.

**Fig. 2:**
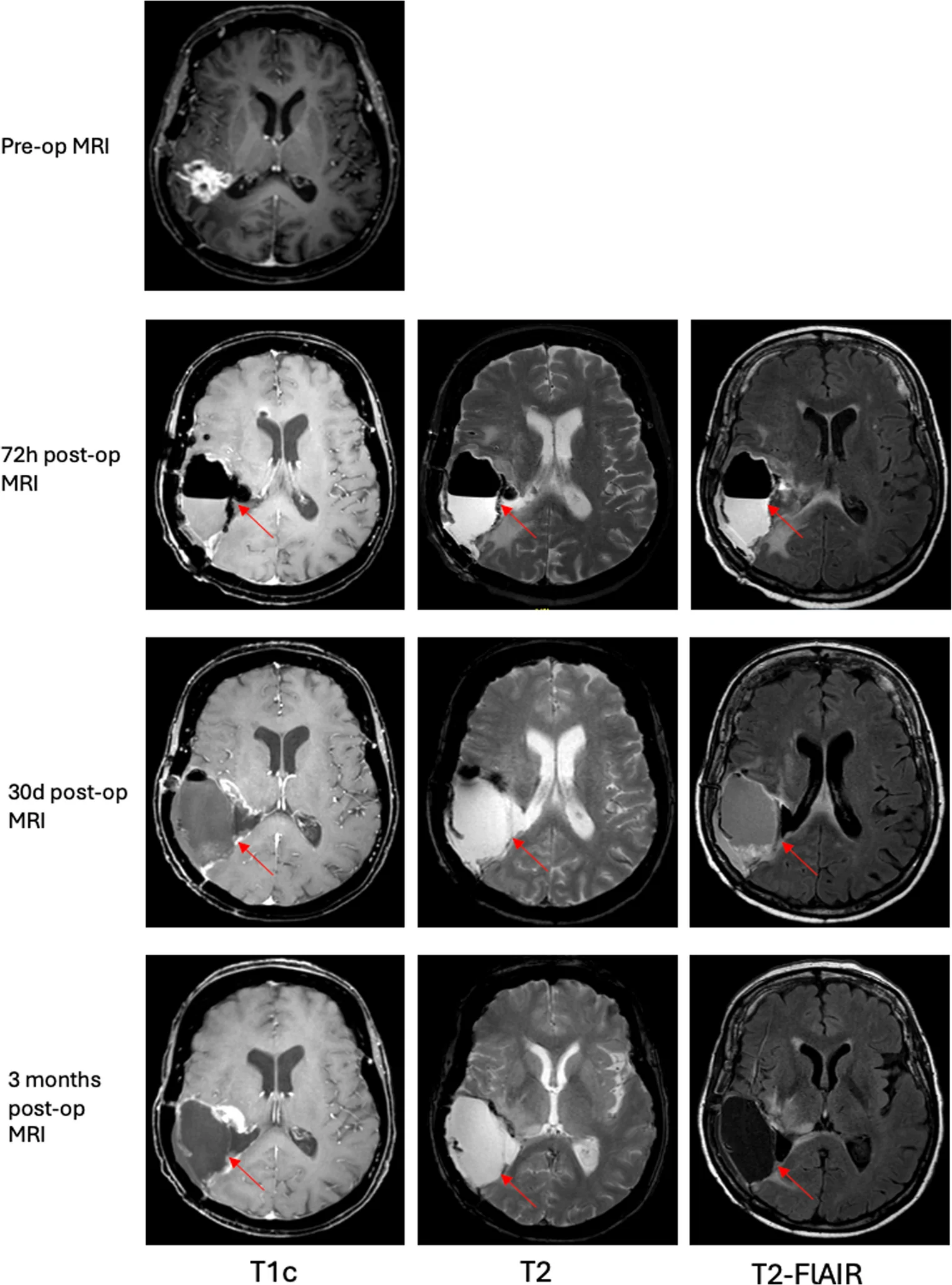
Representative axial MRI of a patient undergoing resection of recurrent glioblastoma with intraoperative ventricular entry and repair of the ependymal defect using TachoSil® (red arrows). The top row shows the preoperative contrast-enhanced T1-weighted (T1c) image, demonstrating an enhancing recurrent right temporoparietal glioblastoma abutting the lateral ventricular system. Postoperative MRI obtained at 72 h, 30 days, and 3 months is shown in the subsequent columns, with T1c, T2-weighted, and T2-FLAIR sequences displayed for each time point. Red arrows indicate the ventricular repair site, which appears as a thin linear interface separating the resection cavity from the ventricular lumen and remains identifiable throughout follow-up, consistent with durable ventricular sealing. No evident imaging features suggestive of transependymal CSF leakage are observed during follow-up

No postoperative meningitis or ventriculitis was observed. Across the cohort, early postoperative MRI showed the expected heterogeneous appearance of the resection cavity, including mixed T1/T2-signal fluid and blood products, dependent sedimentation with fluid–fluid levels, and pericavitary enhancement attributable to postoperative change. This cavity heterogeneity served as an internal comparator for interpreting ventricular signal abnormalities (Fig. 2).

The ventricular CSF space retained predominantly CSF-like signal on non-contrast sequences. When present, intraventricular material was limited to trace dependent layering compatible with minimal blood or proteinaceous content, without evidence of a continuous tract between the resection cavity and the ventricle. At the ventricle–cavity interface, a thin, laminar hypointense band was visible at the site of ventricular entry on early and subsequent MRI, consistent with the expected appearance of a fibrin sealant patch as a discrete layer-like structure, classically markedly hypointense on T1-weighted images and slightly hypointense on T2-weighted images relative to the surrounding soft tissues. When most conspicuous, this interface formed a sharp boundary between the heterogeneous cavity contents and the clean intraventricular CSF, supporting radiological differentiation between surgical-cavity and intraventricular contents (Fig. 2).

Using the early postoperative MRI as the patient-specific baseline, serial review at 1 and 3 months showed that the laminar interface remained centered at the ependymal repair site, without interval displacement, separation, or folding into the ventricular lumen. No new free intraventricular patch-like material, obstruction of the foramen of Monro or other CSF pathways, transependymal CSF flow, progressive ventriculomegaly, or obstructive hydrocephalus was identified. Accordingly, no examination met the retrospective criteria for definite or suspected patch migration through 3 months. The interface remained thin and laminar rather than nodular or progressively mass-like, although differentiation from early tumor regrowth or treatment-related change remains a potential diagnostic challenge. No imaging findings suggestive of ventriculitis, such as frank intraventricular debris with marked diffusion restriction or conspicuous ependymal enhancement, were observed. At the scheduled 3-month outpatient assessment, no additional clinically apparent CSF-related or patch-related complication was documented. Nevertheless, these findings cannot exclude subclinical displacement below MRI resolution or migration occurring after the available follow-up interval.

In all 14 recurrent cases, the prior high-grade glioma resection had not involved ventricular opening; therefore, the ventricular repair interface assessed in this study was newly created during the index procedure. Because these tumors were high-grade, scheduled MRI at 1 and 3 months provided serial assessment of both the repair interface and possible early regrowth or treatment-related change. Interpretation nevertheless remained more challenging in recurrent disease because these findings may alter, mimic, or obscure the resection-margin interface.

### Focused evidence synthesis

Nistor et al. (1997) first reported using a collagen-fibrin patch in skull base surgery for hemostasis and dural repair. In a large series of 288 neurosurgical cases, Reddy et al. (2002) used TachoComb (an earlier form of TachoSil^®^) as a dural substitute, reporting no infections or aseptic meningitis and good fibrous incorporation [34], reporting. Several observational cohorts and technical series then reported low postoperative cerebrospinal fluid (CSF) leak rates when TachoSil^®^ is used as an adjunct to dural or ventricular closure [4, 5, 19, 30, 40, 41].

In elective craniotomy, patch reinforcement may be most appropriate when the dura is friable, retracted, or irregularly lacerated and suturing is unreliable [4]. A randomized double-blind trial reported a 13.5% overall CSF leakage rate; patch augmentation reduced leakage compared with sutures alone (9.7% vs. 17.2%; OR 0.53) without reaching statistical significance, and intermediate care unit stay was shorter in the patch arm [21]. Consistent with a modest incremental benefit when primary watertight dural closure is feasible, a single-center comparative cohort with a systematic review (662 patients; 310 receiving TachoSil^®^) found similar postoperative CSF leak rates with and without patch augmentation (7.74% vs. 7.95%; *p* = 0.960) [4]. Kivelev et al. (2015) tracked 100 microneurosurgical cases where TachoSil^®^ was applied; they reported zero instances of postoperative wound CSF leakage and no TachoSil^®^-related adverse reactions [28]. By final follow-up, 96% of patients had completely uneventful healing, and 4% had minor superficial wound infections (all resolved with oral antibiotics) [28].

In endoscopic endonasal skull base reconstruction, a retrospective series of 121 patients reported a 0.8% postoperative CSF leak rate [22]. Because watertight suturing is often not feasible in this setting, adjunctive patches are commonly incorporated into multilayer reconstructions. In transsphenoidal pituitary surgery, a fibrin-coated collagen patch was associated with lower observed postoperative leak rates than a liquid sealant (7.7% vs. 18.2%; *p* = 0.062) and reduced need for lumbar drainage [2, 40]. Technical series support incorporating TachoSil^®^ into multilayer repairs (e.g., autologous grafts or flaps plus patch), with very low reported leak rates and no clear patch-specific complications [22, 23].

In the spinal compartment, elevated hydrostatic pressures, particularly in the lumbar region, can challenge liquid sealants. Retrospective incidental durotomy series report TachoSil^®^ as effective for small defects, including pinhole tears managed with patch-only repair [5, 16, 43]. Prophylactic patching has also been used when dura is markedly thinned after decompression to reduce delayed rupture and pseudomeningocele formation [16].

Evidence specific to ventricular sealing is limited and largely observational, but available reports support feasibility and an acceptable safety profile in selected periventricular procedures [37, 41, 42]. A milestone case collection specifically addressing ventricular sealing was reported by Bock et al. in 2011 [3]. In this prospective series, 9 patients with malignant gliomas had large intentional ventricular openings (fenestrations > 10 mm) during tumor resection that were occluded with TachoSil^®^ before placing BCNU (Gliadel) wafers [3]. Postoperative imaging showed the TachoSil^®^ fleece as a linear layer separating the resection cavity from the ventricle in all cases, with no wafer migration, hydrocephalus, CSF leakage, or ventricular-entry related neurologic decline during a median 10.4-month follow-up [3]. The authors concluded that sealing the ventricle with TachoSil^®^ effectively separates the resection cavity from the ventricular system and permits safe intra-cavity chemotherapy [3]. The most robust data on ventricular closure with TachoSil^®^ comes from the prospective cohort study by Teixidor-Rodríguez et al. [41]. They enrolled 39 patients undergoing craniotomies for tumors abutting or involving the ventricles and applied TachoSil^®^ to seal any ventricular entry. No adverse drug reactions or septic/aseptic meningitis were observed; CSF dynamics-related complications occurred in 5% and resolved after shunting, and no pseudomeningoceles > 20 cc were reported [41]. Across other neurosurgical cohorts where ventricular sealing is not a primary endpoint, postoperative CSF leak rates with TachoSil^®^ typically range from 1.8% to 7.8%, with comparative studies often showing non-statistically significant trends rather than consistent superiority over alternative sealants [5, 11, 19, 23, 40, 42]. Adjunct sealants and dural substitutes have also been applied around burr holes, shunt sites, and duraplasty in hydrocephalus surgery to mitigate CSF leakage and infection risk, but existing series remain small and heterogeneous [9, 37]. Selected studies relevant to ventricular closure are summarized in Table 1. Compared with these available reports, the present study adds a 32-patient consecutive cohort of WHO grade 3–4 gliomas and represents one of the largest single-center clinical experiences focused on TachoSil^®^ ventricular sealing after glioma resection, with systematic postoperative MRI assessment of the ventricle-cavity interface [3, 41, 42].

Published evidence specifically addressing intraventricular patch migration remains sparse. Bock et al. described TachoSil^®^ as a linear layer separating the resection cavity from the ventricle and reported no patch or wafer migration, hydrocephalus, or ventricular outflow obstruction during a median 10.4-month follow-up [3]. Teixidor-Rodríguez et al. similarly reported no TachoSil^®^-related adverse events during 90-day follow-up in a prospective ventricular-sealing cohort, although that study was not powered to establish the incidence of rare delayed migration [41]. These reports support serial assessment of interface position and morphology together with secondary CSF-dynamics signs, but they do not provide a validated migration-specific MRI protocol or exclude very rare late events [3, 41].

**Table 1:** Representative studies on sealing ventricular openings in neurosurgery. CSF leak = any postoperative cerebrospinal fluid fistula (incisional or rhinorrhea); hydrocephalus = postoperative ventricular enlargement requiring diversion

Study (Year)	Patient Group	Closure Material/Technique	Results – CSF Leak/Hydrocephalus	Other Outcomes
Al-Yamany & Del Maestro (2000) [1]	25 adult pts, transcortical intraventricular tumor resections	Fibrin glue applied to cortical vent tract (no collagen patch)	0% symptomatic subdural CSF collections (no pseudomeningocele)	No mention of infection; first to show glue can prevent fluid leak.
Bock et al. (2011) [3]	9 adult pts, high-grade gliomas with large ventricular entry; BCNU wafers placed	TachoSil^®^ patch covering ventricle opening (prospective case series)	0% postoperative CSF leaks; 0% hydrocephalus; 0% ventricular catheter needed.	No CSF fistula or imaging evidence of ventricular outflow obstruction; no TachoSil^®^-related complications.
Graziano et al. (2016) [20]	30 neurosurgery cases	TachoSil^®^ for hemostasis & reconstruction (incl. ventricle cover in some cases)	“Lower CSF leak rate” with TachoSil^®^ adjunct, but not zero – small number of leaks still occurred (exact rate not specified).	Authors noted TachoSil^®^ helps prevent leaks but does not entirely eliminate them; no TachoSil^®^-related adverse events reported.
Elhabashy et al. (2022) [15]	12 pediatric pts, large intraventricular tumors	Gelfoam sponge plus Tisseel^®^ fibrin glue used to seal ependymal and cortical defect; pia sutures when possible (retrospective)	50% had some postoperative subdural fluid on imaging; 25% (3 patients) developed significant enlarging subdural collections needing intervention (1 subdural shunt; 2 burr-hole drainages).	No meningitis or ventriculitis; no new hydrocephalus post-op (0 VP shunts for hydrocephalus); all improved after managing collections. Highlights that even with meticulous closure, some CSF collections can occur in pediatric large-tumor cases.
Teixidor-Rodríguez et al. (2024) [41]	39 adult pts (40 cranial surgeries) with open ventricle (supratentorial tumors); prospective cohort	TachoSil^®^ placed intraparenchymally as ventricular sealant (single-center study)	5% ventricular-related complications: 1 case (2.5%) of external CSF leak and 1 case (2.5%) of external hydrocephalus; *no* pseudomeningocele. Both patients improved with VP shunt; 0% required re-operation for leak.	0 infections and 0 adverse reactions in 90-day follow-up. No TachoSil^®^-related problems observed. Authors conclude TachoSil^®^ is a safe & effective ventricular sealant, though call for randomized trials.

## Discussion

### Interpretation of the present findings and technical considerations

This retrospective series supports the technical feasibility and short-term safety of an onlay TachoSil^®^ patch for ventricular closure during supratentorial high-grade glioma resection. No patient developed meningitis or ventriculitis, shunt-dependent hydrocephalus, early reoperation for suspected patch-related obstruction, permanent CSF diversion, or definite radiological migration. Serial MRI showed predominantly CSF-like intraventricular signal and a stable thin interface at the repair site, consistent with restoration of a functional barrier between the resection cavity and ventricular system. These findings do not demonstrate comparative efficacy, because no untreated or alternatively treated control group was included and the study was underpowered for rare complications. Patient selection, operative technique, dural closure quality, tumor location, and the natural history of ventricular-entry glioma surgery may also have influenced outcomes.

The observed repair stability is biologically and mechanically plausible. TachoSil^®^ combines a pliable collagen matrix with human fibrinogen and thrombin; contact with moist tissue produces a fibrin network that adheres the fleece and mechanically interlocks with the underlying surface [4, 10, 18, 25]. In ventricular applications, it is best used as a flat lid bridging the ependymal defect rather than packed into the lumen. Adequate overlap, correct active-surface orientation, uninterrupted initial compression, and minimal manipulation are important because insufficient overlap or displacement before fibrin fixation may compromise sealing [13, 18, 25, 27, 30, 31, 38, 41, 42, 44]. The absence of folding, displacement, free intraventricular material, or CSF-pathway obstruction supports stable initial fixation. However, defect size, overlap, compression time, and high-flow leak characteristics were not recorded systematically, precluding analysis of technical predictors of sealing or postoperative collections.

### CSF-related outcomes and comparison with the literature

The main unfavorable finding was a 15.6% rate of CSF-related subcutaneous collection or wound leakage. This exceeded the 0% postoperative leak reported by Bock et al. [3], the 5% ventricular CSF-dynamics complication rate reported by Teixidor-Rodríguez et al. [41], and the approximately 7.7%–7.9% leak rates in mixed cranial cohorts with or without TachoSil^®^ augmentation [4, 12]. Severity was limited: four cases resolved with compression and observation, one required temporary lumbar drainage, and none required wound revision, permanent diversion, or reoperation. Comparisons should remain cautious because all patients had WHO grade 3–4 gliomas and 43.8% underwent surgery for recurrent disease, where previous surgery and adjuvant treatment may impair tissue quality and healing. Ventricular defect dimensions, leak-flow characteristics, dural closure, and reinforcement were also not standardized. As imaging showed no persistent cavity–ventricle communication, progressive ventriculomegaly, or patch displacement, some collections may have reflected dural or wound-level factors rather than ependymal repair failure. This remains inferential without a control group and standardized characterization of ventricular and dural defects.

### Safety, imaging interpretation, patch migration and and CSF-dynamics surveillance

The absence of infection, aseptic meningitis, clinically apparent inflammation, or ventricular obstruction is consistent with the favorable safety profile reported for fibrin-coated collagen patches in cranial and ventricular applications [5, 13, 18, 19, 24, 28, 30, 36, 41]. Preclinical and histopathological evidence suggests progressive tissue infiltration and remodeling rather than persistent mass formation, although rare foreign-body or granulomatous reactions remain possible and require longer surveillance [13, 18, 24, 31]. Imaging interpretation is particularly important in high-grade and recurrent glioma [39]. In the 14 recurrent cases, previous surgery had not involved ventricular entry, so the observed interface was attributable to the index repair. The thin laminar band remained centered at the ependymal defect on MRI within 72 h and at 1 and 3 months. Persistent linear morphology favored expected patch appearance, whereas nodular or progressively mass-like change would suggest recurrence. Early regrowth and treatment-related enhancement may nevertheless mimic or obscure the interface, so radiologists should know the implantation site.

Migration is a relevant theoretical concern because detached material could obstruct a narrow CSF pathway and cause delayed hydrocephalus. Review therefore assessed direct signs—loss, discontinuity, folding, displacement, or new free intraventricular patch-like material—and indirect signs, including foramen of Monro or other CSF-pathway obstruction, transependymal flow, progressive ventriculomegaly, and hydrocephalus. None occurred through 3 months. This agrees with Bock et al., who reported a persistent linear layer without migration, hydrocephalus, or outflow obstruction over a median 10.4 months [3], and with the 90-day experience of Teixidor-Rodríguez et al. [41]. A fixed collagen-fibrin patch with limited swelling may offer advantages over liquid sealants that spread before polymerization or hydrogels that expand in confined CSF-adjacent spaces [6, 25, 33, 44]. However, no validated migration-specific MRI protocol exists, routine MRI may miss subtle displacement, and bioresorption may reduce patch conspicuity. The findings therefore support only short-term safety and cannot exclude subclinical or delayed migration within the limitations of this retrospective, uncontrolled study design.

## Strengths

The study focuses on a specific and clinically relevant scenario–ventricular entry during supratentorial glioma resection–rather than pooling heterogeneous dural closure indications. Consecutive inclusion of treated cases reduces selection bias within the included cohort, and the technique is described in sufficient operative detail to support reproducibility. The combination of clinical CSF-dynamics endpoints with serial MRI assessment provides a practical description of the postoperative ventricular repair interface.

## Limitations and future directions

The evidence on TachoSil^®^ in cranial and skull base surgery includes randomized trials, prospective cohorts, systematic reviews, and predominantly retrospective series, but remains limited by small, single-center samples, selection bias, incomplete data capture, residual confounding, and few randomized comparisons [2, 4, 10, 14, 16, 17, 19, 23, 26, 29, 33, 36, 38, 40–42]. Evidence specific to ventricular closure is mainly observational and heterogeneous in endpoint definitions, follow-up, surgical technique, adjunct materials, application parameters, and adverse-event reporting, limiting comparability and meta-analytic pooling [14, 18, 23, 25, 28, 30, 33, 35–38, 40, 41, 44, 45].

These limitations also apply to the present small, single-center, uncontrolled retrospective series. No contemporaneous or historical institutional control group or reliable denominator for untreated ventricular openings was available, and the cohort was underpowered to detect rare events such as delayed patch migration, obstructive hydrocephalus, or granulomatous reaction. Clinical follow-up was more intensive during the first 30 postoperative days and included a scheduled outpatient clinical assessment at 3 months, with MRI available at 1 and 3 months. However, systematic clinical follow-up beyond 3 months was not available, and later complications may therefore have been missed. Ventricular defect size, high-flow leak features, dural closure technique, and use of dural reinforcement were not standardized, limiting risk stratification and potentially confounding CSF-related wound outcomes. Although recurrent disease in this cohort followed prior high-grade glioma surgery without previous ventricular opening and serial MRI at 1 and 3 months allowed temporal assessment of the newly created repair interface, treatment-related changes or early regrowth could still mimic or obscure the patch appearance. Although restriction to WHO grade 3–4 gliomas reduced histological heterogeneity, it limits generalizability to lower-grade tumors.

Future prospective multicenter studies should include appropriate comparison cohorts, longer follow-up, and standardized reporting of ventricular defect characteristics, leak classification, dural closure details, intraoperative testing, migration-focused imaging, and late adverse events. Harmonized protocols, including initiatives such as ENCASE, are needed to improve between-study comparisons and support robust future meta-analyses [4, 7, 16, 25, 30, 35–38, 40, 44, 45].

## Conclusions

Available evidence and this exploratory cohort suggest that onlay TachoSil^®^ ventricular sealing is technically feasible and did not reveal short-term meningitis/ventriculitis, shunt-dependent hydrocephalus, early reoperation for obstruction, or definite radiological patch migration in this selected series. However, the study cannot establish comparative efficacy, the 15.6% CSF-related subcutaneous collection or wound leakage rate warrants caution, and rare or delayed complications cannot be excluded. Prospective comparative studies with standardized reporting of ventricular defect size, dural closure variables, high-flow leak features, migration-focused MRI findings, and longer clinical follow-up are required before stronger clinical recommendations can be made.

## Data Availability

No datasets were generated or analysed during the current study.

## Declarations

### Patient and/or carer perspective

Patient- or carer-reported narratives, interviews, or quotations were not collected because this was a retrospective analysis of de-identified routinely collected data and the reported ventricular-sealing strategy was performed as part of standard operative management.
